# Volumetric lattice Boltzmann method for wall stresses of image-based pulsatile flows

**DOI:** 10.1038/s41598-022-05269-w

**Published:** 2022-02-01

**Authors:** Xiaoyu Zhang, Joan Gomez-Paz, Xi Chen, J. M. McDonough, Md Mahfuzul Islam, Yiannis Andreopoulos, Luoding Zhu, Huidan Yu

**Affiliations:** 1 Department of Mechanical and Energy Engineering, Indiana University-Purdue University, Indianapolis (IUPUI), Indianapolis, IN 46202 USA; 2 Department of Mechanical Engineering, The City College of New York, New York, NY 10031 USA; 3 Departments of Mechanical Engineering and Mathematics, University of Kentucky, Lexington, KY 40506 USA; 4 Department of Mathematical Sciences, Indiana University-Purdue University, Indianapolis (IUPUI), Indianapolis, IN 46202 USA; 5 Department of Vascular Surgery, Indiana University School of Medicine, Indianapolis, IN 46202 USA

**Keywords:** Engineering, Mathematics and computing

## Abstract

Image-based computational fluid dynamics (CFD) has become a new capability for determining wall stresses of pulsatile flows. However, a computational platform that directly connects image information to pulsatile wall stresses is lacking. Prevailing methods rely on manual crafting of a hodgepodge of multidisciplinary software packages, which is usually laborious and error-prone. We present a new computational platform, to compute wall stresses in image-based pulsatile flows using the volumetric lattice Boltzmann method (VLBM). The novelty includes: (1) a unique image processing to extract flow domain and local wall normality, (2) a seamless connection between image extraction and VLBM, (3) an en-route calculation of strain-rate tensor, and (4) GPU acceleration (not included here). We first generalize the streaming operation in the VLBM and then conduct application studies to demonstrate its reliability and applicability. A benchmark study is for laminar and turbulent pulsatile flows in an image-based pipe (Reynolds number: 10 to 5000). The computed pulsatile velocity and shear stress are in good agreements with Womersley's analytical solutions for laminar pulsatile flows and concurrent laboratory measurements for turbulent pulsatile flows. An application study is to quantify the pulsatile hemodynamics in image-based human vertebral and carotid arteries including velocity vector, pressure, and wall-shear stress. The computed velocity vector fields are in reasonably well agreement with MRA (magnetic resonance angiography) measured ones. This computational platform is good for image-based CFD with medical applications and pore-scale porous media flows in various natural and engineering systems.

## Introduction

Pulsatile flows are omnipresent in a wide range of engineering and scientific systems. Examples include pulmonary ventilating^1,2^ and blood circulating^3^ in biological flows, sediment transport in coastal flows^4^, and reciprocating flow in internal combustion engines^5^. Understanding turbulence in pulsatile flows is critically important to both knowledge advancement and technological innovation but challenges have remained due to the complexities of these flows. A typical pulsatile flow is by nature four-dimensional (4-D: 3-D in space plus 1-D in time). It is composed of a positive mean and a periodically varying time-dependent component around the mean. It is inherently unsteady with successive flow acceleration and deceleration, resulting in timewise transitions from laminar to turbulent flow (turbulentization) and then back to laminar flow (relaminarization) during pulsation^6,7^. Since the seminal work of Reynolds ^8^, significant efforts have been made to study the transition to turbulence in pulsatile flows^9–13^. The focus has been on the exploration of criteria for laminar, transitional, and turbulent flow in pulsatile flows from different perspectives including hydrodynamic stability^14^ and instability^15^, pulsatility^16^, and turbulence dynamics^17^. All these studies were for pulsatile flows in pipes. Pulsatile flow is commonplace in the cardiovascular system, and its flow domain geometry is far more complicated than a straight pipe. The wall stresses connect the blood flow dynamics and the vessel wall response, providing pathophysiological insights for different cardiovascular diseases that are not readily measurable in medical practice. Wall-normal stress (WNS) is associated with blood pressure, leading to the deformation of cells in the vessel wall, whereas wall-shear stress (WSS) acts on the endothelium of the vessel wall through a shearing deformation (‘shearing force’). The wall stresses play central roles, not only in aneurysm initiation, growth, and rupture^18^ but also in the development of atherosclerosis^19^. With the advances in medical imaging, scientific modeling, and computational power, image-based computational fluid dynamics (ICFD) has emerged^20–26^ as a new computer-aided tool for diagnostics and therapeutics of cardiovascular diseases. Based on medical imaging data, such as computed tomography angiogram (CTA) or magnetic resonance angiogram (MRA), together with Doppler ultrasound sonography (DUS), ICFD has enabled noninvasive evaluation of 4-D in vivo vectorial velocity and pressure in the entire arterial system with fine spatial and temporal resolution. For an arbitrarily oriented wall, both WNS and WSS are vectors, projected from the total stress tensor on local normal and tangential directions of the wall, respectively, with magnitudes and directions (derivation shown later). Full characterization of the wall stresses for cardiovascular hemodynamics includes its topological, spatiotemporal, and vectorial nature^19,27^, which remains challenging in ICFD due to the fact that the real human vasculature is usually irregular in geometry and orientation with curvatures and bifurcations. When the vessel is diseased with either stenosis (lumen reduction) or aneurysm (lumen enlargement), its geometry can be exceptionally complicated.

A general process of ICFD for wall stresses mainly involves four tasks: (1) *extraction* of flow domain and local normal orientation from image data, (2) *connection* between output of image processing and input of CFD, (3) *quantification* of 4-D velocity and pressure fields employing physical parameters together with initial and boundary conditions using CFD, and (4) *calculation* of wall stresses via post-processing. Either commercial software or an open-source package that directly solves ICFD from image data to wall stresses is currently unavailable. The prevailing approach for ICFD is to utilize a hodgepodge of multidisciplinary software and/or open-source packages, such as Materialise Mimics, ScanIP, BLOD3D, Avizo, and 3D VIEWNIX for image segmentation and ANSYS, COMSOL, STAR-CD, OpenFOAM, and FEFLOW for CFD. Since the packages for image processing and CFD are independent, one needs to use additional software and/or open-source packages to connect these two tasks via geometry rescaling and mesh generation. The representative software and open-source packages for such connection are T-grid, Pointwise, Mesh lab, Gmesh, and Meshfix. Although popular, such a process is usually laborious and error-prone due to the need for manual crafting of each software and/or open-source package. Meanwhile, in conventional CFD, the strain-rate tensor, also shear stress, is calculated from the 1^st^-order derivatives of the velocity vector field as part of the post-processing. Total stress tensor and wall stresses (WSS and WNS) are then calculated. There are two challenges in this part of the post-processing. The first is the necessity of determining the normal direction on the local wall, which is difficult for a regular cubic mesh with a Cartesian coordinate system when the flow domain is irregular and complicated. The second is that the velocity adjacent to the wall is small and noisy, especially when the flow is turbulent. Computing near-wall velocity requires fine resolution, which can cause a substantial computational burden. In fact, the computational cost of ICFD for pulsatile flows is inherently high and will become demanding when the flow is turbulent.

In this work, we present a unique computational platform for quantifying 4-D wall stresses in image-based pulsatile flows using the volumetric lattice Boltzmann method (VLBM)^28^ to address the aforementioned challenges. The novelty of this computational approach consists of (1) a unique extraction of flow domain and local wall normality from image data, (2) a seamless connection between the output of image processing and the input of CFD, (3) an en-route calculation of strain-rate tensor for wall stresses, and (4) GPU parallel computing processing^29–32^ (not included in this paper) to significantly mitigate the computation burden. The kinetic-based lattice Boltzmann modeling has emerged for simulating a broad class of complex flows including pore-scale porous media flow^33–37^, multiphase/multicomponent flows^38–42^, and turbulence^40,43–47^. The main advantages related to this work are its amenability for modeling the intermolecular interactions at the two-phase interface to recover the appropriate multiphase dynamics without demanding computing cost and its suitability for scalable GPU (Graphics Processing Unit) parallelization^29,31,32,40,43–48^ to achieve fast computation. The VLBM was specially developed for dealing with arbitrarily complicated flow domains, e.g., porous media and blood capillaries, with or without moving boundaries. In this work, we generalize the streaming operation from its original formulation^28^.

Aiming toward broad applications for not only biomedical flows but also parametric-designed flows in nature and engineering, we start with STL (Standard Triangle Language) data format for images in this computational platform. Medical images from CTA or MRA are in DICOM (Digital Imaging and Communications in Medicine) format. Image segmentation is required to extract the anatomical vessel before the vessel geometry is fed to the VLBM for hemodynamics. The raw DICOM images usually involve noises from different resources and cleaning up the noises is always a critical part of the tasks for an image segmentation to extract the anatomical vessels. Such a task is usually case to case with a lot of uncertainties, related to the imaging modality, machine resolution, scanning skills, disease conditions, etc. Although we have developed techniques for DICOM image segmentation^30,49,50^, we strongly recommend having rigorous image segmentation with expertise, instead of simply running software with extensive manual inputs, as it will affect the quantification of the hemodynamics. All the modern CAD (Computer-Aided Design) software packages such as SolidWorks export their native file format into STL format. The conversion from DICOM segmentation to an STL format^51^ is straightforward.

We conduct two studies to demonstrate the reliability and applicability of the computational method. The first is a systematic benchmark study of pulsatile flows in an image-based pipe. We simulate Womersley (laminar) flows and compare the 4-D computed fields of the velocity, shear stress tensor, and WSS with analytical solutions. Then we simulate turbulent flows varying the base flow Reynolds number (defined later) from 535 to 4375 and compare the computed 4-D velocity and WSS with experimental data. The second is an application study to quantify 4-D hemodynamics in MRA-based human cerebral and carotid arteries.

The remainder of the paper is organized as follows. In “Volumetric lattice Boltzmann modeling and formulation for wall stresses in ICFD”, we present the computational method including the VLBM, the image processing, the connection of both, and the en-route calculation of WSS and WNS. A benchmark study of pulsatile flows, both laminar and turbulent flows, in an image-based pipe and an application study of blood flow in MRA-based human arteries are carried out in “Benchmark study of oscillating flows in a 3-D pipe” and “Application study”, respectively. Finally, “Summary and discussion” concludes the paper with a summary and discussion.

## Volumetric lattice Boltzmann modeling and formulation for wall stresses in ICFD

**Figure 1:**
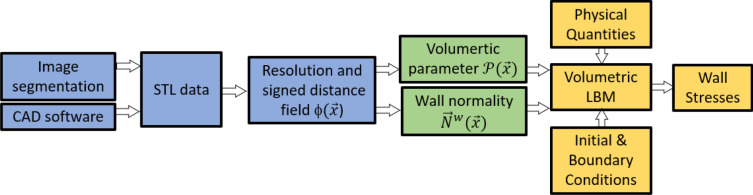
Flowchart from image data to wall stresses. The volumetric parameter and wall normality (green) are the outputs of image processing (blue) and inputs of CFD using VLBM (yellow), enabling seamless connection between both.

As schematized in Fig. 1, the VLBM for wall stresses in ICFD consists of three components: image acquisition and segmentation (blue), determination of volumetric parameter and wall normality (green), and CFD using VLBM for wall stresses (yellow). Image segmentation and CFD are seamlessly connected as the volumetric parameter and wall normality are the outputs of the former and the inputs of the latter. In this section, we present the modeling and formulation from three aspects: the formulation of volumetric LBEs, geometrical processing, and en-route calculation of wall stresses. It is noted that the streaming operation in the LBEs will be generalized from its original formula^28^.

### Formulation of volumetric lattice Boltzmann method

**Figure 2:**
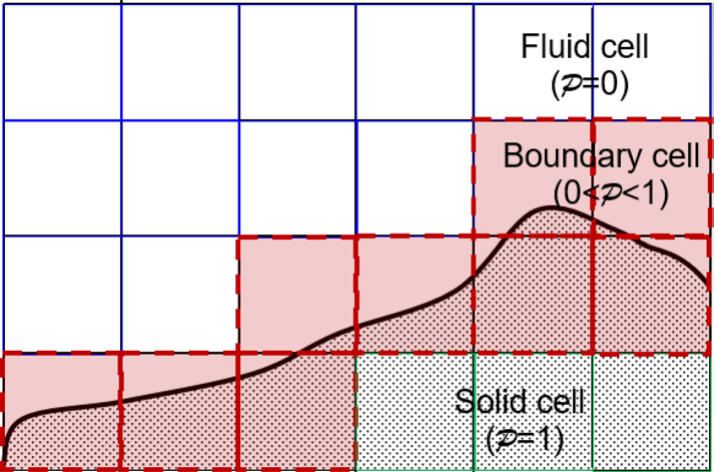
Illustration of the cell-based lattice involving solid ($\mathcal{P}=1$, green), fluid ($\mathcal{P}=0$, white), and boundary (0 < $\mathcal{P}$  < 1, red) cells.

The VLBM^28^ was developed specifically for treating arbitrarily oriented boundaries with or without boundary movement. In the VLBM, fluid particles are uniformly distributed in lattice cells. By introducing a volumetric parameter $\mathcal{P}\left(\vec{x},t\right)$, defined as the occupation of solid volume in the cell, i.e.,$\mathcal{P}\left(\vec{x},t\right)\equiv \Delta {V}_{s}\left(\vec{x},t\right)/\Delta V\left(\vec{x},t\right)$ , we distinguish three types of lattice cells in the simulation domain: solid cell (pure solid occupation, $\mathcal{P}$ = 1), fluid cell (pure fluid occupation, $\mathcal{P}$ = 0), and boundary cell (partial solid and partial fluid, 0 < $\mathcal{P}$  < 1), as illustrated in Fig. 2.

Formulation of the VLBM is self-regularized to deal with the complex flow geometries through $\mathcal{P}$. It consists of three operations: (1) collision considering the momentum exchange between the moving boundary and the flow; (2) streaming accompanying a volumetric bounce-back procedure in boundary cells; and (3) migrating volumetrically moving the residual fluid particles into the flow domain when the boundary swipes over a boundary cell toward a solid cell. The VLBM strictly satisfies mass conservation and can handle any irregular boundary orientation and motion with respect to the mesh. In this work, we consider fixed flow geometries only, thus $\mathcal{P}\left(\vec{x},t\right)=\mathcal{P}\left(\vec{x}\right),$ which is a one-time calculation from STL image data. Thus, the collision reverts to the conventional operation in LBM and the migrating operation is omitted.

We use the D3Q19 lattice model to formulate the volumetric LBEs. The 19 discrete molecular velocities are

$${\vec{e}}_{i}=\left\{\begin{array}{cccc}(\mathrm{0,0},0)& & & i=0\\ (\pm \mathrm{1,0},0)c,& (0,\pm \mathrm{1,0})c,& (\mathrm{0,0},\pm 1)c& i=1-6\\ (\pm 1,\pm \mathrm{1,0})c,& (\pm \mathrm{1,0},\pm 1)c,& (0,\pm 1,\pm 1)c& i=7-18\end{array}\right.$$

where $\mathrm{c }(=\frac{\delta x}{\delta t}$ with $\delta x$ and $\delta t$ the lattice width and time interval respectively) represents the lattice speed in lattice units. We select $\mathrm{c}=1$, i.e., $\delta x=\delta t=1$, meaning that the particles stream one lattice unit per time step. The sound speed of the D3Q19 model in the lattice units is ${c}_{s}=\frac{c}{\sqrt{3}}$; and the weighting factors ${\omega }_{i}$ are ${\omega }_{0}=\frac{1}{3}$, ${\omega }_{1-6}=\frac{1}{18}$, and ${\omega }_{7-18}=\frac{1}{36}$.

The VLBM deals with the time evolution of the particle population in each lattice cell, and the volumetric LBE with BGK (Bhatnagar, Gross, and Krook) approximation^52^ for the collision operation reads

1 $${n}_{i}\left(\vec{x}+{\vec{e}}_{i}\delta t,t\right)-{n}_{i}\left(\vec{x},t\right)=-\frac{1}{\tau }\left[{n}_{i}\left(\vec{x},t\right)-{n}_{i}^{eq}\left(\vec{x},t\right)\right]+{F}_{i}\left(\vec{x},t\right), i=0\sim 18$$

where ${n}_{i}\left(\vec{x},t\right)$, ${n}_{i}^{eq}\left(\vec{x},t\right)$, $\tau$, and ${F}_{i}\left(\vec{x},t\right)$ are the particle population, the equilibrium particle population, the relaxation time due to fluid-particle collisions, and the forcing term, respectively, in cell $\vec{x}$ at time *t*. The forcing term is formulated^44,53^ as

2 $${F}_{i}\left(\vec{x},t\right)=-3{\omega }_{i}N\frac{{\vec{e}}_{i}\cdot \vec{a}\left(\vec{x},t\right)}{{c}^{2}}\delta t$$

with $\vec{a}$ the acceleration due to an external force. The equilibria for incompressible flows are

3 $${n}_{i}^{eq}\left(\vec{x},t\right){=N\omega }_{i}\left\{1+\frac{3{\vec{e}}_{i}\cdot \vec{u}}{{c}^{2}}+\frac{9{({\vec{e}}_{i}\cdot \vec{u})}^{2}}{2{c}^{4}}-\frac{3\vec{u}\cdot \vec{u}}{2{c}^{2}}\right\}$$

where $$N\left(\vec{x},t\right)\equiv \sum_{i=0}^{18}{n}_{i}\left(\vec{x},t\right)\equiv \sum_{i=0}^{18}{n}_{i}^{eq}\left(\vec{x},t\right)$$ and $$N\left(\vec{x},t\right)\vec{u}\left(\vec{x},t\right)\equiv \sum_{i=0}^{18}{{\vec{e}}_{i}n}_{i}\left(\vec{x},t\right)\equiv \sum_{i=0}^{18}{{\vec{e}}_{i}n}_{i}^{eq}\left(\vec{x},t\right)$$.

The relation between the particle density distribution function ${f}_{i}\left(\vec{x},t\right)$ in a node-based LBM and the particle population ${n}_{i}\left(\vec{x},t\right)$ in cell-based VLBM is ${n}_{i}\left(\vec{x},t\right)=[1-\mathcal{P}\left(\vec{x}\right)]{f}_{i}(\vec{x},t)$. Since $\Delta {V}_{f}\left(\vec{x},t\right)=\left[1-\mathcal{P}\left(\vec{x}\right)\right]\Delta V,$ ${n}_{i}\left(\vec{x},t\right)$ collapses to ${f}_{i}\left(\vec{x},t\right)\text{ in fluid cells where }\mathcal{P}=0$.

The implementation of Eq. (1) includes two operations. The first involves the collision operator, ${\Omega }_{i}\left(\vec{x},t\right)$, resulting in ${n{^{\prime}}}_{i}\left(\vec{x},t\right)$ that is referred to as ‘post-collision’ particle population.

4 $${n{^{\prime}}}_{i}\left(\vec{x},t\right){=n}_{i}\left(\vec{x},t\right)-\frac{1}{\tau }\left[{n}_{i}\left(\vec{x},t\right)-{n}_{i}^{eq}\left(\vec{x},t\right)\right]+{F}_{i}\left(\vec{x},t\right)$$

**Figure 3:**
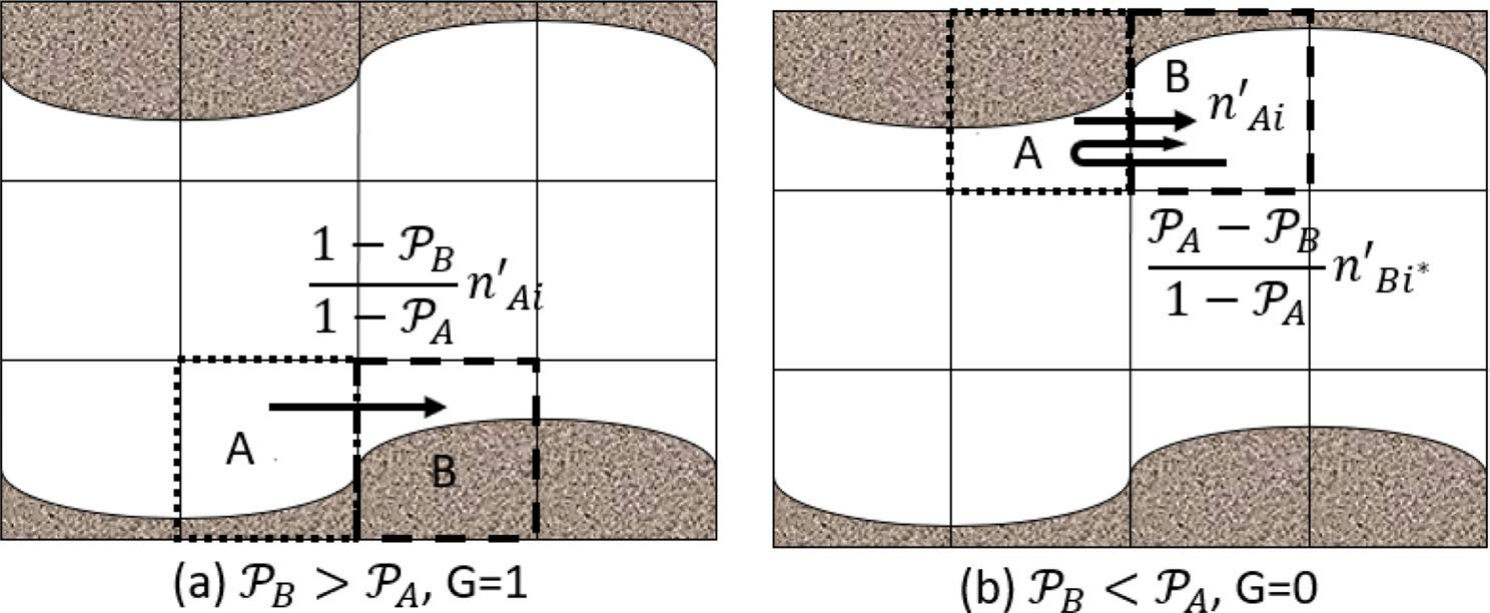
Illustration of two streaming scenarios when the current cell B at $\vec{x}$ (dashed) and upwind cell A (dotted) at $\vec{x}+{\vec{e}}_{i*}\delta t$ are both boundary cells. A parameter G is introduced to distinguish these two cases. (**a**) ${\mathcal{P}}_{B}>{\mathcal{P}}_{A}$, G = 1. The receiving particles are streamed from the upwind cell. Because the fluid fraction of B is smaller than that of cell A, only $\frac{1-{\mathcal{P}}_{B}}{1-{\mathcal{P}}_{A}}{n{^{\prime}}}_{Ai}$ particles of cell A can stream into cell B and no bounced-back particles. (**b**) ${\mathcal{P}}_{B}<{\mathcal{P}}_{A}$, G=0. The receiving particles consists of two parts: streamed from the upwind cell A (*n*′_*Ai*_) and the bounced-back particles from the current cell $\frac{{\mathcal{P}}_{A}-{\mathcal{P}}_{B}}{1-{\mathcal{P}}_{B}}{n{^{\prime}}}_{Bi}$∗ in the opposite direction.

The second operation is for streaming, which reflects the particles moving from the current cell to the neighboring cells. Since a boundary cell is partially occupied by fluid, only a partial volume fraction of fluid particles can stream to a neighboring cell in the direction of the molecular velocity. In the original paper^28^, the streaming equation is formulated as follows.

5 $${n}_{i}(\vec{x},t+\delta t)=[1-\mathcal{P}(\vec{x})]\cdot {n{^{\prime}}}_{i}\left(\vec{x}-{\vec{e}}_{i}\delta t,t\right)+\mathcal{P}(\vec{x}+{\vec{e}}_{i*}\delta t)\cdot {n{^{\prime}}}_{i*}\left(\vec{x},t\right)$$

where ${i}^{*}$ is the particle velocity direction opposite to the *i*-th direction, i.e., ${\vec{e}}_{i*}=-{\vec{e}}_{i}$ . In Eq. (5), the first term is the streaming term. All the particles in the upwind neighboring cell, ${n{^{\prime}}}_{i}\left(\vec{x}-{\vec{e}}_{i}\delta t,t\right),$ stream to the current cell but the current cell can only accept $[1-\mathcal{P}(\vec{x})]\cdot {n{^{\prime}}}_{i}\left(\vec{x}-{\vec{e}}_{i}\delta t,t\right)$. This part assumes that the upwind cell is a fluid cell. The second term is the bounce-back term. $[1-\mathcal{P}(\vec{x}+{\vec{e}}_{i*}\delta t)]\cdot {n{^{\prime}}}_{i*}\left(\vec{x},t\right)$ particles stream to the upwind neighboring cell from the current cell and $\mathcal{P}(\vec{x}+{\vec{e}}_{i*}\delta t)\cdot {n{^{\prime}}}_{i*}\left(\vec{x},t\right)$ particles bounce back to the current cell. This part assumes that the current cell is a fluid cell. However, either the current or the neighboring cell can be a boundary cell, with which Eq. (5) becomes problematic because the capability to stream and/or receive particles is determined by the relative fluid volume between the two cells. Also, Eq. (5) does not apply for the scenario that both current and upwind cells are boundary cells with an equal fluid fraction. To address these deficiencies, we consider two scenarios in the streaming operation as illustrated in Fig. 3. Assume cell B, located at $\vec{x}$, is receiving particles during the streaming, and cell A is cell B’s upwind neighboring cell located at $\vec{x}+{\vec{e}}_{i*}\delta t$. If ${\mathcal{P}}_{B}>{\mathcal{P}}_{A}$ , see Fig. 3a, the receiving particles are purely streamed from cell A. Because the fluid fraction of cell B is smaller than that of cell A, only $\frac{1-{\mathcal{P}}_{B}}{1-{\mathcal{P}}_{A}}{n{^{\prime}}}_{Ai}$ particles among the total particles in cell A in the *i*-th direction can stream into cell B and the remaining particles $\frac{{\mathcal{P}}_{B}-{\mathcal{P}}_{A}}{1-{\mathcal{P}}_{A}}{n{^{\prime}}}_{Ai}$ will bounce back to cell A in the direction of ${\vec{e}}_{i*}$. Figure 3b illustrates another scenario, ${\mathcal{P}}_{B}<{\mathcal{P}}_{A}$, in which the receiving particles are from two sources: ${n{^{\prime}}}_{Ai}$(streamed from cell A) and $\frac{{\mathcal{P}}_{A}-{\mathcal{P}}_{B}}{1-{\mathcal{P}}_{A}}{n{^{\prime}}}_{B{i}^{*}}$ (bounced back from ${n{^{\prime}}}_{B{i}^{*}}$). We now introduce parameter G as

6 $${G}_{i}(\vec{x})=\left\{\begin{array}{c} \text{ 1, if }\mathcal{P}\left(\vec{x}+{\vec{\mathrm{e}}}_{\mathrm{i}*}\delta t\right.)<\mathcal{P}\left(\vec{x}\right.), \\ \text{ 0, if }\mathcal{P}\left(\vec{x}+{\vec{\mathrm{e}}}_{\mathrm{i}*}\delta t\right.)\ge \mathcal{P}\left(\vec{x}\right.)\end{array}\right.$$

and generalize Eq. (5) as

7 $${n}_{i}\left(\vec{x},t+\delta t\right)={G}_{i}(\vec{x})\frac{1-\mathcal{P}\left(\vec{x}\right)}{1-\mathcal{P}\left(\vec{x}+{\vec{e}}_{i*}\delta t\right.)}{n}_{i}\left(\vec{x}+{\vec{e}}_{i*}\delta t,t\right)+\left[1-{G}_{i}\left(\vec{x}\right)\right]\left[{n}_{i}\left(\vec{x}+{\vec{e}}_{i*}\delta t,t\right)+\frac{\mathcal{P}\left(\vec{x}+{\vec{e}}_{i*}\delta t\right.)-\mathcal{P}(\vec{x})}{1-\mathcal{P}(\vec{x})}{n}_{i*}\left(\vec{x},t\right)\right]$$

In the case of ${\mathcal{P}}_{B}={\mathcal{P}}_{A}$, Eq. (7) becomes ${n}_{i}\left(\vec{x},t+\delta t\right)={n}_{i}\left(\vec{x}+{\vec{e}}_{i*}\delta t,t\right)$, which Eq. (5) cannot achieve.

The resulting density, velocity, and pressure of the flow system are obtained as follows:

8 $$\uprho \left(\vec{x},t\right)=\frac{{\sum }_{i=0}^{18}{n}_{i}\left(\vec{x},t\right)}{1-\mathcal{P}(\vec{x})}$$

9 $$\vec{u}\left(\vec{x},t\right)=\frac{{\sum }_{i=0}^{18}{{\vec{\mathrm{e}}}_{\mathrm{i}}n}_{i}\left(\vec{x},t\right)}{{\sum }_{i=0}^{18}{n}_{i}\left(\vec{x},t\right)}$$

and

10 $$p\left(\vec{x},t\right)-{p}_{0}={c}_{s}^{2}[\rho \left(\vec{x},t\right)-{\rho }_{0}]$$

where ${p}_{0}$ and ${\rho }_{0}$ (= 1) are reference pressure and density in lattice units, respectively.

The LBM can handle arbitrary oriented boundaries with or without motion, and it satisfies mass conservation strictly. The formulation taking into consideration of bounce-back boundary conditions is self-regulated by the volumetric parameter $\mathcal{P}(\vec{x})$ that distinguishes fluid, boundary, and solid cells. The implementation of the volumetric LBEs only occurs in fluid and boundary cells. Thus, the key to the VLBM is the determination of $\mathcal{P}\left(\vec{x}\right)$ for a flow domain.

### Geometrical processing for volumetric parameter $\mathcal{P}\left(\vec{{\varvec{x}}}\right)$ and local wall normality ${\vec{N}}^{w}(\vec{x})$

Geometrical processing is an important step in ICFD needed to connect image segmentation to CFD. Usually, it requires extra effort for geometry rescaling and mesh generation. When using the VLBM as the CFD solver, a seamless connection between the image information and CFD can be achieved by quantifying the volumetric parameter $\mathcal{P}\left(\vec{x}\right)$ for the entire simulation domain. The normal direction of each boundary cell, ${\vec{N}}^{w}(\vec{x})$, which is required for calculating WSS and WNS, is calculated concurrently. The geometrical process from STL data to the volumetric parameter $\mathcal{P}(\vec{x})$ and wall normality ${\vec{N}}^{w}(\vec{x})$ is seen in Fig. 1. The green part is the output of the STL image processing (blue part) and the input of VLBM (yellow part), enabling a seamless connection between image information and CFD. The geometrical process consists of three main components described in the following subsections.

### Level set method for signed-distance field $f(\vec{x})$ from STL data

**Figure 4:**
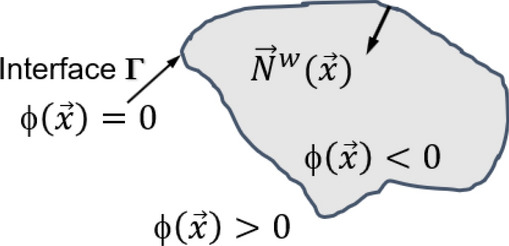
Signed distance field $\phi \left(\vec{x}\right)$: $\phi \left(\vec{x}\right)=0$ locates on the interface Γ with inward normal ${\vec{N}}^{w}(\vec{x})$ . $\phi \left(\vec{x}\right)<0$ and $\phi \left(\vec{x}\right)>0$ mean inside and outside of surface Γ.

The level set method is a conceptual framework for using level sets as a tool for numerical analysis of surfaces and shapes. One can perform numerical computations involving curves and surfaces on a fixed Cartesian grid without having to parameterize these objects^54^. A signed-distance function $\phi (\vec{x}$) determines the distance of a given point $\vec{x}$ from an interface, with the sign determined by whether $\vec{x}$ is inside or outside the interface. When a flow domain is closed and bounded by an interface *Γ* with inward normal ${\vec{N}}^{w}$, as shown in Fig. 4, the signed distance field $\phi \left(\vec{x}\right)$ represents the geometric space using a level set of

11 $$\phi \left(\vec{x}\right)= \left\{\begin{array}{c}>0, \quad \vec{x}\in \Gamma \\ =0, \quad \vec{x} \text{ on } \Gamma \\ <0, \quad \vec{x}\notin \Gamma \end{array}\right.$$

with

$$|\nabla \phi |=1$$

The STL data describes a raw, unstructured triangulated surface by the unit normal and vertices (ordered by the right-hand rule) of the triangles using a 3-D Cartesian coordinate system. This file format is supported by many other software packages, e.g., MATLAB. We use existing open-source packages of *READ_stl.m*, *VOXELISE.m*^55^, and *ac-reinit.m*^56^ to read the STL file, generate a 3-D uniform mesh with the specified resolution, and calculate the signed distance field $\phi \left(\vec{x}\right)$ in MATLAB.

### Volumetric parameter $\mathcal{P}\left(\vec{x}\right)$ and wall-normal direction ${\vec{N}}^{w}(\vec{x})$ from signed distance field $\phi \left(\vec{x}\right)$

**Figure 5:**
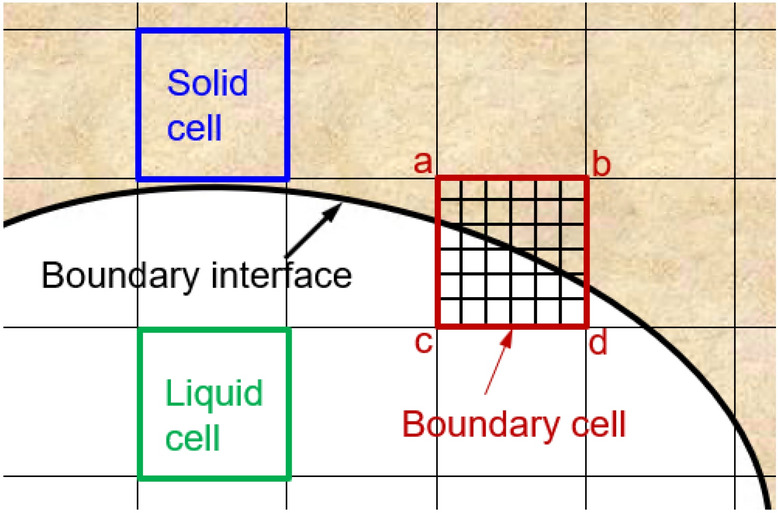
Identification of fluid cell, solid cell, and boundary cell based on the $\phi$ signs at four vertexes of each cell. All negative $\phi$s mean a fluid cell (green, $\mathcal{P}=0$), all positive $\phi$s means a solid cell (blue, $\mathcal{P}=1$), and partial negative (vertexes c and d) partial positive (vertexes a and b) indicates a boundary cell (red,$0< \mathcal{P}<1$).

For an illustration, we use a 2-D lattice, in Fig. 5, to describe how to determine the $\mathcal{P}$ value for a cell from the signed distance field $\phi \left(\vec{x}\right)$. Given the interface, which is the boundary of the flow domain, the sign $\phi$ at each node of the lattice indicates the area, either fluid (white) or solid (orange), and the node location. If the $\phi$s at the four vertices of a cell are all negative, the cell is inside the fluid domain, and $\mathcal{P}=0$; whereas, if all four $\phi$s are positive, the cell is in the solid domain and $\mathcal{P}=1$, see green and blue cells in Fig. 5, respectively. Otherwise, the cell is a boundary cell (red) occupied by partial solid and partial fluid.

For a boundary cell, we use the following steps to calculate the $\mathcal{P}$ value.

- Uniformly divide the boundary cell into ${q}^{2}$ sub-cells. Each cell is represented by the square center ${\vec{x}}_{s}^{j}$ with a solid volume ${V}_{s}^{j}$, $j=1,\dots ,{q}^{2}.$
- Calculate the $\phi$ value at its ${\vec{x}}_{s}^{j}$ using the known $\phi$ values at 4 vertices of the cell through linear interpolation for each sub-cell. If $f({\vec{x}}_{s}^{j})>0$, set ${V}_{s}^{j}=1$; otherwise, set ${V}_{s}^{j}=0.$
- Obtain the total solid volume through a summation of the solid volume of all the ${q}^{2}$ sub-cells: ${V}_{s}={\sum }_{j=1}^{{q}^{2}}{V}_{s}^{j}\left({\vec{x}}_{s}^{j}\right)$.
- Obtain the solid volume fraction, i.e., the $\mathcal{P}$ value, as $\mathcal{P}={V}_{s}/{q}^{2}$.

Note that more sub-cells, equivalent to larger *q*, result in a more accurate $\mathcal{P}$ value, but more computation will be needed to calculate the volume fraction. Thus, *q* should be appropriately chosen to balance between accuracy and computational cost.

The inward normal direction at the volumetric center of a boundary cell can be easily determined. The normal direction to the boundary wall is needed to calculate the WSS and WNS, see Eqs. (17) and (18) below. For a 3-D flow domain, a boundary cell is divided into ${q}^{3}$ sub-cells, each of which is identified by its geometrical center ${\vec{x}}_{s}^{j}$ and its volume ${V}_{s}^{j}\left({\vec{x}}_{s}^{j}\right)$. Notice that ${V}_{s}^{j}\left({\vec{x}}_{s}^{j}\right)$ is either 1 if positive $\phi ({\vec{x}}_{s}^{j})$ or 0 if negative $\phi ({\vec{x}}_{s}^{j})$. The volumetric center of the fluid in a boundary cell can be determined by ${\vec{x}}_{b}=\frac{{\sum }_{j=1}^{{q}^{3}}[1-{V}_{s}^{j}\left({\vec{x}}_{s}^{j}\right)]{\vec{x}}_{s}^{j}}{{\sum }_{j=1}^{{q}^{3}}[1-{V}_{s}^{j}\left({\vec{x}}_{s}^{j}\right)]}$ . Then based on the volumetric center ${\vec{x}}_{b}$ and the distance functions $\phi$s at the eight vertices of the cubic cell, linear interpolation results in the direct distance of the volumetric center to the boundary surface.

The normal direction of the local interface in each boundary cell can be obtained from the gradient of the distance function at the eight vertices. We use ${\vec{x}}_{ver}^{j}$ with $j=1,\dots ,8$ to denote a vertex’s location. With the available distance field $\phi \left(\vec{x}\right)$, the gradient $\nabla \phi ({\vec{x}}_{ver}^{j})$ results in a vector indicating the normal direction and the steepness of the local boundary surface. Using a linear interpolation from the distance of ${\vec{x}}_{ver}^{j}$ with $j=1,\dots ,8$ to the ${\vec{x}}_{b}$, we get the $\nabla \phi ({\vec{x}}_{b})$. The normal direction of the boundary cell can then be determined as follows

12 $${\vec{N}}^{w}({\vec{x}}_{b})=\frac{\nabla \phi ({\vec{x}}_{b})}{\left|\nabla \phi ({\vec{x}}_{b})\right|}$$

### En-route calculation of wall stresses

In hydrodynamics, the strain-rate tensor is defined as ${S}_{\alpha \beta }\equiv \frac{1}{2}\left(\frac{\partial {u}_{\alpha }}{\partial {x}_{\beta }}+\frac{\partial {u}_{\beta }}{\partial {x}_{\alpha }}\right)$, where $\alpha (=1, 2, 3)$ and $\beta (=1, 2, 3)$ represent the indices of the axial directions in a 3-D Cartesian coordinate system. We use these notations in the LBM, it can be calculated from the non-equilibrium distribution function as ^57^

13 $${S}_{\alpha \beta }=-\frac{1}{2\rho \tau {c}_{s}^{2}}{\sum }_{i}{e}_{i\alpha }{e}_{i\beta }\left({f}_{i}-{f}_{i}^{eq}\right)$$

It has been demonstrated that Eq. (13) is more robust than the conventional finite difference method (FDM) to calculate the strain rate from the velocity field^44^.

In the VLBM, Eq. (13) becomes

14 $${S}_{\alpha \beta }=-\frac{1}{2N\tau {c}_{s}^{2}}{\sum }_{i}{e}_{i\alpha }{e}_{i\beta }\left({n}_{i}-{n}_{i}^{eq}\right)$$

Observing that shear stress tensor ${\sigma }_{\alpha \beta }=2\mu {S}_{\alpha \beta }$, pressure ${p=c}_{s}^{2}\frac{N}{1-\mathcal{P}}$, and the viscosity $\mu ={c}_{s}^{2}\left(\tau -\frac{1}{2}\right)$, we can formulate the total stress tensor as

15 $${T}_{\alpha \beta }=-\frac{N{c}_{s}^{2}}{(1-\mathcal{P})}{\delta }_{\alpha \beta }+\left(2\tau -1\right){c}_{s}^{2}{S}_{\alpha \beta }$$

where ${\delta }_{\alpha \beta }$ is the Kronecker unit tensor.

The Cauchy formula gives the overall stress on the wall with a normal vector ${\vec{N}}^{w}$:

16 $${T}_{\alpha }^{(W)}={T}_{\alpha \beta }{N}_{\beta }^{w}$$

in which Einstein summation convention with index notation has been used. Then its projection onto the normal direction yields the WNS

17 $${T}_{\alpha }^{(WNS)}={(N}_{\beta }^{w}{T}_{\gamma \beta }{N}_{\gamma }^{w}) {N}_{\alpha }^{w}$$

Here $\gamma (=\mathrm{1,2},3)$ is also an index for the axial direction in the 3-D Cartesian coordinate system. The WSS is then computed as the difference between the overall stress and its projection onto the normal:

18 $${T}_{\alpha }^{(WSS)}={T}_{\alpha \beta }{N}_{\beta }^{w}-({n}_{\beta }{T}_{\gamma \beta }{n}_{\gamma }){n}_{\alpha }$$

With the determination of the local wall normality ${\vec{N}}^{w}$ in Eq. (12), both WSS and WNS (when needed) can be obtained en-route during the VLBM implementation.

## Benchmark study of oscillating flows in a 3-D pipe

We now apply the VLBM to simulate pulsatile laminar and turbulent flows in an image-based 3-D pipe, focusing on demonstrating the reliability and applicability of the computational platform by comparing the computational results with analytical and experimental results.

### Computation set-up

A 3-D rigid, right-circular straight pipe is generated in *Solidworks* in STL format with a length L = 19.05 mm and a radius R = 9.525 mm. The pulsatile flow along the pipe in the z-direction is driven by either a pressure gradient, $P\left(t\right)$ or a velocity profile, ${\vec{u}}_{in}(x,y,t)$, which will be specified below. A no-slip boundary condition is applied on the pipe wall, realized by the bounce-back boundary condition. It is noted that in VLBM, the bounce-back boundary condition is involved in the streaming operation, see Eqs. (6) and (7), regulated by the volumetric parameter $\mathcal{P}\left(\vec{x}\right)$. The initial conditions are constant pressure and zero velocity in the entire flow domain. A uniform mesh in the VLBM is used with the cell number, ${N}_{D}$, across the pipe diameter to represent the spatial resolution.

The volumetric parameter $\mathcal{P}\left(\vec{x}\right)$ of each lattice cell and the normal direction of each boundary cell ${\vec{N}}^{w}(\vec{x})$ are calculated based on the STL file by our in-house MATLAB code. As mentioned in “[Sec Sec6]”, when the volumetric parameter $\mathcal{P}\left(\vec{x}\right)$ of a boundary cell at location $\vec{x}$ is calculated, the boundary cell is divided into ${q}^{3}$ sub-cells and $q$ should be appropriately chosen to balance between accuracy and computation cost. We perform an analysis on this theme. First, we evaluate the influence of the spatial resolution represented by ${N}_{D}$ through the relative error, ${\delta }_{V}$ (%), of the $\mathcal{P}\left(\vec{x}\right)$-based fluid volume (summation of $1-\mathcal{P}\left(\vec{x}\right)$ over the entire computation domain) from the specified pipe volume ($2\pi {R}^{2}L$). Table 1 shows, when $q=8$ is selected, the error reduces to below 1% when ${N}_{D}$ =127. We then stick to ${N}_{D}=127$ to study the effects of *q* on the computation time and accuracy of $\mathcal{P}\left(\vec{x}\right)$ in Table 2. It is seen that when *q* increases, the computation time increases significantly but the accuracy remains almost the same. Since our general goal for accuracy is less than 1%, we use $q=8$ to compute the $\mathcal{P}\left(\vec{x}\right)$ field with ${N}_{D}>127$ in what follows. The wall-clock time was recorded on a computer with Inter®Xeon® CPU E5-1650 v3 @ 3.50 GHz, RAM 32 GB, 64-bit Operating System, ×64-based processor, and Matlab R2020a.

**Table 1:** Influence of spatial resolution ${N}_{D}$ on the accuracy of $\mathcal{P}\left(\vec{x}\right)$ at $q=8$.

${N}_{D}$	65	91	127
δ_*V*_ (%)	1.8	1.3	0.35

**Table 2:** Effects of *q* on the computation time and accuacy of $\mathcal{P}(\vec{x})$ at *N*_*D*_=127.

*q*	4	8	12	16
Wal-clock time (s)	203	244	344	588
δ_*V*_ (%)	0.35	0.34	0.34	0.34

### Laminar pulsatile flows

In this section, we simulate a Womersley (laminar) flow driven by a pulsatile pressure gradient, $P(t)={P}_{s}+Re\left({P}_{o}{e}^{i\omega t}\right)$. We use ${P}_{s}={P}_{0}=0.02Pa/m$ and $\omega =6.28{s}^{-1}$. The pressure gradient is introduced as a body force (external force) ${F}_{i}$ in Eq. (1) via Eq. (2) where $\vec{a}(t)=\frac{P(t)}{\rho }\vec{k}$ along the z-direction. With the body force to drive the flow, we impose a periodic boundary condition along z-direction. The density and kinematic viscosity of the fluid is 1.0e3 kg/m^3^ and 1.0e-6 m/s^2^, respectively.

**Figure 6:**
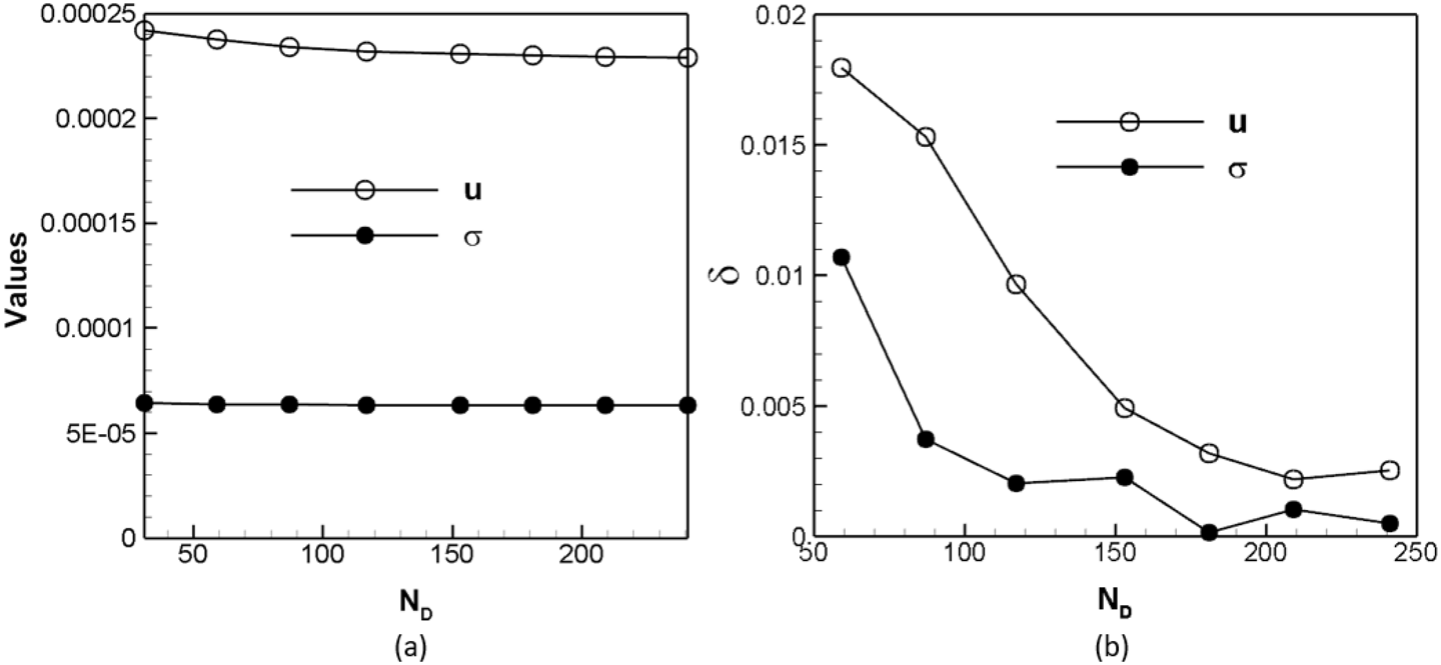
Convergence checks for determining appropriate spatial resolution: (a) Spatial mean velocity u (empty circle) and shear stress σ (solid circle) and (b) relative differences of *u* and σ between two successive resolutions (δ) versus the cell number along the diameter (${N}_{D}$).

We first perform a grid-function convergence check to determine the appropriate spatial resolution. Seven ${N}_{D}$s of 31, 59, 87, 117, 153, 181, 209, and 241 are used. Figure 6a shows the effects of *N*_*D*_ on the spatial mean downstream velocity $u$ and mean shear stress σ on a cross-section. It is seen that each of the curves becomes flat as ${N}_{D}$ increases. The quantitative measurement of the convergence is shown in Fig. 6b, in which the convergence rate $\delta$ is calculated as the relative difference of the quantity of interest—velocity or shear stress—between two successive spatial resolutions. It indicates that when ${N}_{D}$ ≥150, $\delta <0.5\%$ for both velocity and shear stress. For the simulations of laminar and turbulent flows below, *N*_*D*_ is in the range of 150–240. Correspondingly, the relaxation time *τ* is in the range of 0.55–0.95.

### Order of accuracy

The order of accuracy of a finite-difference-related numerical scheme is usually evaluated by the discretization error. As pointed out in Ferziger’s textbook^58^, the exact solution of the discretized equations on the grid $h(=\frac{2R}{{N}_{D}})$, ${\varphi }_{h}$, differs from the exact solution of the partial differential equation,$\varPhi$, by the discretization error ${\delta }_{h}$, i.e., ${\delta }_{h}=\varPhi -{\varphi }_{h}$. For sufficiently fine grids, this discretization error is proportional to the leading term in the Taylor series: ${\delta }_{h}\approx \alpha {h}^{q}+H$ where $H$ stands for higher-order terms and $\alpha$ depends on the derivatives at the given point but is independent of $h$. The discretization error can be estimated if solutions on systematically refined grids are compared through the expression $\varPhi ={\varphi }_{h}+\alpha {h}^{b}+H={\varphi }_{3h}+\alpha {(3h)}^{b}+H$ with 3 times (for the cell-based cubic mesh) for the increase of reference spacing from $h$. The exponent $b$ represents the order of the numerical scheme and can be estimated as follows

19 $$b=\frac{log\left(\frac{{\varphi }_{3h}-{\varphi }_{9h}}{{\varphi }_{h}-{\varphi }_{3h}}\right)}{\mathrm{log}3}$$

We simulated the flow with three successive resolutions with ${N}_{D}=$ 31, 93, 279. Using the velocity magnitude at three locations r/R = 0, 0.5, and 0.86, we calculate the orders of accuracy for the three locations using Eq. (19) and obtained $b=1.2, 1.2,$ and 1.1, respectively, which indicates that the spatial accuracy of VLBM is 1.2 in interior and 1.1 at the boundary. It is well-known that the spatial accuracy of node-based LBM is theoretically no more than the second order in the interior. The accuracy reduces to the first order near the boundary with a bounce-back boundary condition. When the wall lies exactly at the nodes or middle of two neighboring nodes (half-way bounce-back).

### Computational results vs. analytical solutions of Womersley flow

**Figure 7:**
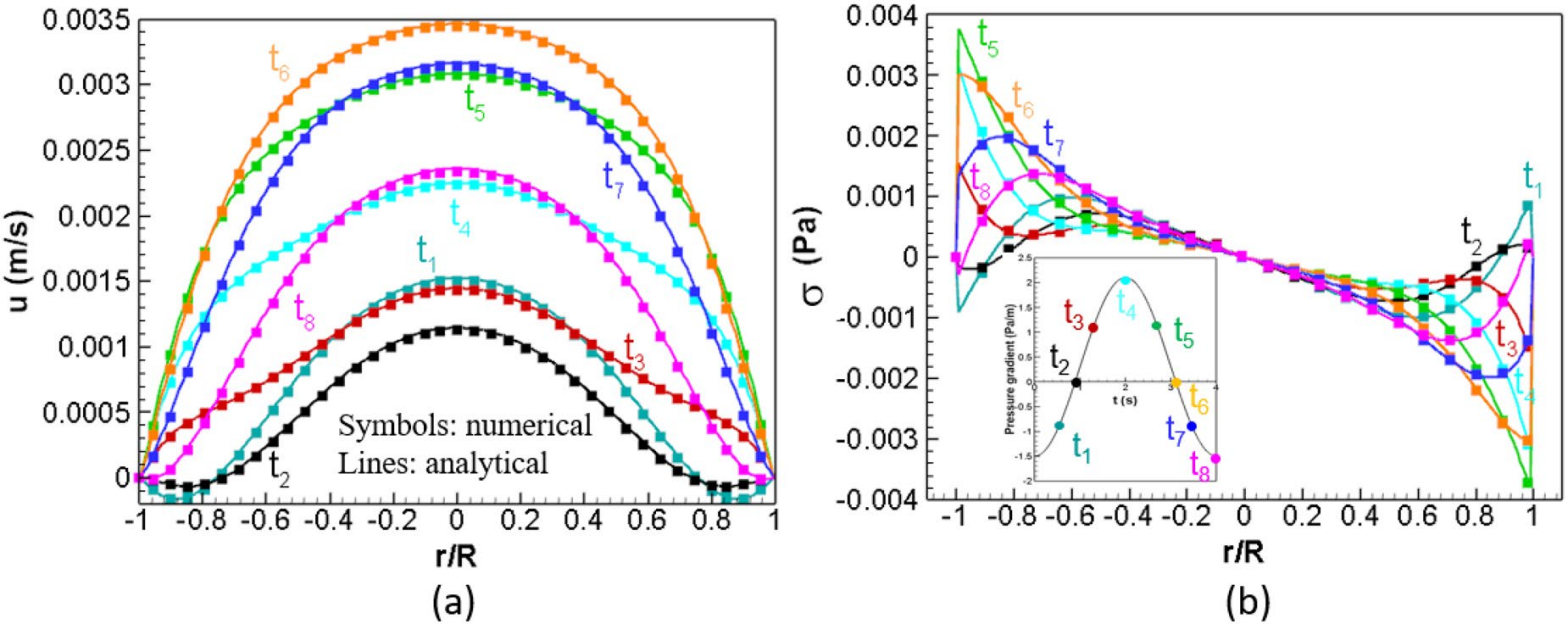
Comparisons of numerical results (symbols) and analytical solutions (lines) at 8 representative time instants, shown in the embedded plot, in a pulsation for velocity u **(a)** and shear stress σ **(b)**, respectively. The Womersley number $\alpha$ and the base flow Reynolds number ${Re}_{0}$ of the flow are 6.89 and 9.4, respectively.

We use the analytical solutions of Womersley flow^59^ to verify the computational results. A Womersley flow, driven by a pulsatile pressure gradient, $P={P}_{s}+{P}_{o}{e}^{i\omega t},$ has the following analytical solutions of downstream velocity:

20 $$u\left(\mathrm{r},t\right)=\frac{{P}_{s}{R}^{2}}{4\mu }\left(1-\frac{{r}^{2}}{{R}^{2}}\right)+\mathrm{Re}\left\{\frac{{P}_{o}{R}^{2}}{i\mu {\alpha }^{2}}\left[1-\frac{{J}_{0}(\frac{\alpha r}{R}{i}^\frac{3}{2})}{{J}_{0}(\alpha {i}^\frac{3}{2})}\right]{e}^{i\omega t}\right\}$$

where *u* is the downstream velocity, ${J}_{0}(\cdot )$ is the Bessel function of the first kind and order zero, $Re(\cdot )$ is the real part of a complex function, $\alpha (=R\sqrt{\frac{\omega }{n}})$ is the Womersley number, and $r(=\sqrt{{x}^{2}+{y}^{2}}$) is the distance to the center of a cross-section. The strain-rate, $S=\frac{\partial u}{\partial r},$ shear stress $\sigma =2\mu S$, and WSS ${\sigma }_{w}={\sigma |}_{wall}$ can be calculated from Eq. (20). Keeping the same pipe configuration, we use ${P}_{s}=0.3 Pa$, ${P}_{o}=1.8 Pa$, and $\omega =1.57 {s}^{-1}$ to simulate the flow in this part. The Womersley number $\alpha$ and the base flow Reynolds number ${Re}_{0}=\frac{2R{u}_{0}}{n}$, with ${u}_{0}$ the spatial mean velocity and $\nu (=\frac{\mu }{\rho })$ the kinematic viscosity, of the flow, are 6.89 and 9.4, respectively. Figure 7 compares the simulation results (symbols) with the analytical solutions (lines) at 8 representative time instants in a pulsation for the downstream velocity *u* (a) and shear stress σ (b) and good agreements between them are shown. More quantitative comparisons are presented in Table 3 with the relative difference between the numerical results and analytical solutions, calculated by $\sqrt{\frac{\sum_{i,j}{({f}_{num}-{f}_{ana})}^{2}}{\sum_{i,j}{f}_{ana}^{2}}}$ where $f$ is for *u* and ${\sigma }_{w}$(WSS) on a cross-section of the pipe at the same representative time instants as embedded in Fig. 7b. Besides, we compared the strain rate (not shown) calculated from en-route VLBM implementation and post-processing of velocity field using FDM. The VLBM is more accurate than the 3-point FDM but nearly equal accurate between VLBM and 5-point FDM.

**Table 3:** Relative errors calculated by $\sqrt{\frac{\sum_{i,j}{({f}_{num}-{f}_{ana})}^{2}}{\sum_{i,j}{f}_{ana}^{2}}}$ where $f$ is for u and ${\sigma }_{w}$ on a cross-section of the pipe at the same representative time instants as embedded in Fig. 7b. The average relative errors at the 8 time points are 1.53% for velocity and 3.98% for WSS. The analytical solution of ${\sigma }_{w}$ at t_8_ tends to zero thus the relative error is not available.

	t_1_	t_2_	t_3_	t_4_	t_5_	t_6_	t_7_	t_8_
$u$	2.42%	2.38%	1.12%	1.06%	1.09%	1.15%	1.29%	1.68%
${\sigma }_{w}$	5.16%	6.57%	4.30%	2.96%	2.42%	2.36%	4.15%	$N/A$

Last but not the least, we demonstrate how Eq. (7) improves the modeling of the generalized streaming operation in the VLBM. Keeping everything else the same, we use Eqs. (5) and (7) to solve the velocity field. The accuracy of the spatial mean velocity comparing with the analytical solutions from Eq. (20) at the 8 time-points in one cycle is presented in Table 4. The average relative error is reduced to 0.653% (Eq. (7)) from 0.721% (Eq. (5)).

**Table 4:** Relative errors calculated by $\sqrt{\frac{\sum_{i}{({u}_{num}-{u}_{ana})}^{2}}{\sum_{i}{u}_{ana}^{2}}}$, where *u* is the velocity at the centerline of cross section of a pipe at the same representative time. The average relative errors at 8 time points are 0.721% for the original equation, Eq. (5), and 0.653% for the generalized equation, Eq. (7).

	t_1_	t_2_	t_3_	t_4_	t_5_	t_6_	t_7_	t_8_
Equation (5)	0.717%	0.709%	0.713%	0.716%	0.724%	0.732%	0.728%	0.726%
Equation (7)	0.649%	0.642%	0.647%	0.650%	0.657%	0.663%	0.660%	0.658%

### Turbulent pulsatile flows

We now validate the reliability and accuracy of the numerical simulation for more realistic pulsatile flows, relatively high base flow Reynolds number ${Re}_{0}$, by comparing velocity and WSS waveforms with laboratory measurements^60^. Time-resolved particle image velocimetry (PIV) techniques were used to acquire two components of velocity vector data on the plane of laser illumination that included the longitudinal axis of an acrylic pipe. The optical system consisted of a single frequency continuous-wave AR + laser, model Spectra-Physics Millennia Vs of 5.5 W with continuous output power at 532 nm. A fast frame-rate CMOS camera, Vision Research v710 Phantom, was used to acquire flow images with a 1200 × 800 pixel resolution. In each experiment, 20,000 images were acquired with a rate of 1 kHz. Hollow glass particles of 9–13 μm diameter (type borosilicate glass spheres LaVision 110P8) were used as tracers to visualize the flow. The PIV images were processed using an in-house modified code based on the open-source PIVLab software for MATLAB platforms. This code is a multi-pass PIV FFT (fast Fourier transform)-correlation based algorithm. The initial interrogation size was 128 × 128 pixels. It is reduced to a final window size of 16 × 16 pixels during three iterations in order to improve the signal-to-noise ratio. Five different experiments were carried out with ${Re}_{0}$ in the range 535 to 4825 and α from 11.91 to 23.82.

**Figure 8:**
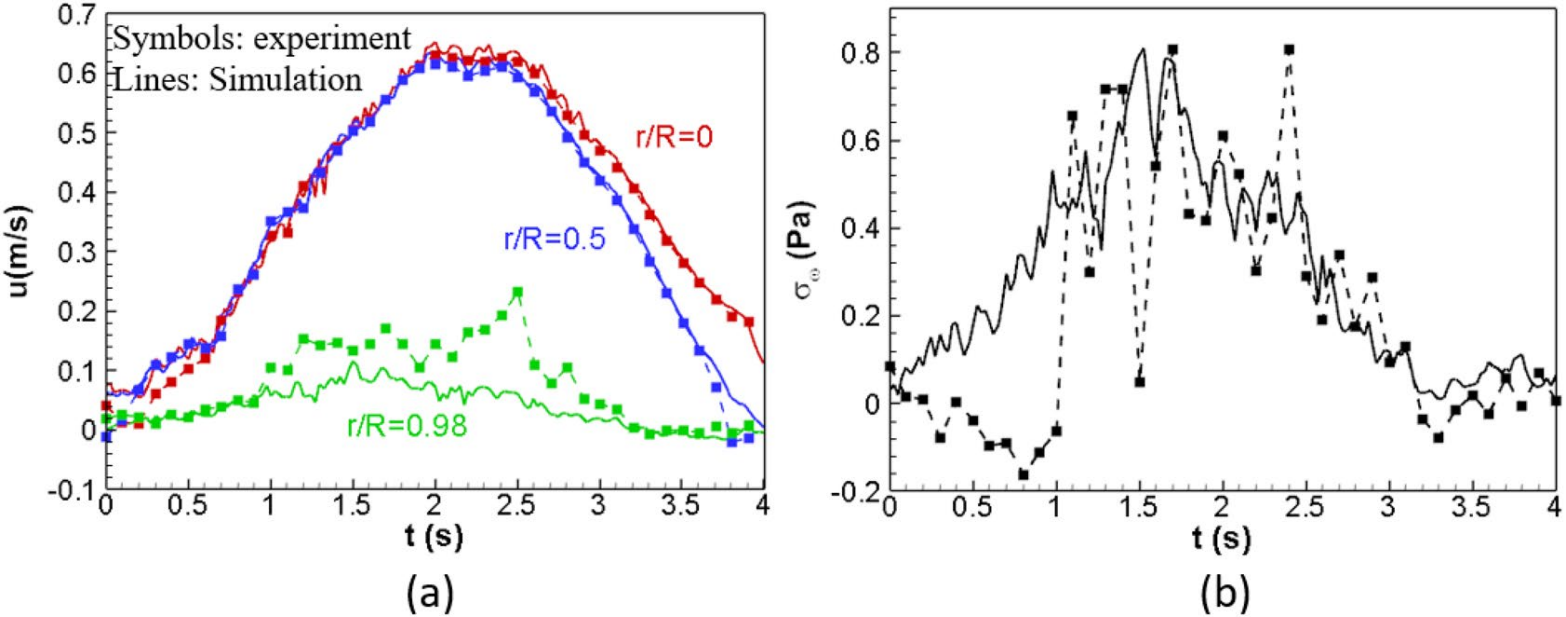
Comparisons of **(a)** velocity waves at the center ($r/R=0$), middle location ($r/R=0.5$), and near boundary ($r/R=0.98$) and **(b)** WSS wave between numerical simulation (lines) and experimental measurement (symbols) with ${Re}_{0}=4735$ and $\alpha =11.91.$

We simulated all the five flows along the downstream region from location 183 to 184.55 in the pipe via direct numerical simulation. The diameter of the pipe (19.05 mm) and the fluid properties, i.e., density ($998.008 \mathrm{kg}/{\mathrm{m}}^{3}$) and kinematic viscosity ($1.004\times {10}^{-6}\mathrm{m}/{\mathrm{s}}^{2}$), are identical to the experiment. In the simulating region, velocity has been measured at 61 downstream locations. A velocity profile at each location contains velocity information at 39 spatial locations along the pipe diameter and 400 temporal samples during a pulsatile period, i.e., 0.25 Hz. We use the velocity profiles at downstream locations at 183 and 184.55 as the inflow and outflow boundary conditions, respectively. They are introduced in the VLBM via a non-equilibrium extrapolation scheme^61^. We use ${N}_{d}=243$ and $\tau =0.506$ for direct numerical simulations of turbulence. The spatial–temporal velocity information at each location is introduced in the D3Q19 lattice cell model on the corresponding cross-section via piecewise cubic Hermite interpolation. The comparisons of (a) velocity waves at the center (r/R = 0), middle location (r/R = 0.5), and near the boundary (r/R = 0.98) and (b) WSS wave between numerical simulation (lines) and experimental measurement (symbols) for the case of ${Re}_{0}=4735$ and $\alpha =11.91$ are shown in Fig. 8. The WSS representing the experimental results in Fig. 8b was calculated from the experimentally measured velocity fields via a 5-point forward FDM. Figure 8a shows that the simulated velocity waves in the inner pipe at $r/R=0$ and $0.5$, agree with the experimental measurements very well. Whereas at the inner wall of the pipe ($r/R=0.98$), both velocity (Fig. 8a) and WSS (Fig. 8b) waves from simulation still agree with the experimental measurements, but the simulation results are consistently less noisy than those from experiments.

## Application study

Beyond the benchmark study, we now apply the presented computational method to a medical research project to explore the capability of non-invasive quantification of 4-D hemodynamics in image-based human vessels. Our focus is the pulsatile velocity, pressure, and WSS in human vertebral and carotid arteries based on MRA.

Cardiovascular disease is known to be strongly influenced by hemodynamic WSS. Although the advanced diagnostic imaging modalities such as digital subtraction angiography, CTA, ultrasound, and MRA can provide very good information of the vessel morphology and the total blood flow, they cannot measure the WSS, which is believed to play a pertinent role in the development of human vascular diseases as demonstrated in many studies^62–65^. Without the quantitative physiological parameters of the flow and blood-vessel interaction, it remains challenging to fully assess the true risk of vascular lesions to optimize treatment decisions. In this project, we aim to integrate patient-specific ICFD and WSS analysis into diagnostic MRA of vascular diseases focusing on carotid arteries. To demonstrate the reliability and applicability of the VLBM-based ICFD for realistic flow systems, we present one study case for the accuracy of the computed hemodynamics including velocity and WSS in human vertebral and carotid arteries following the flowchart in Fig. 1.

**Figure 9:**
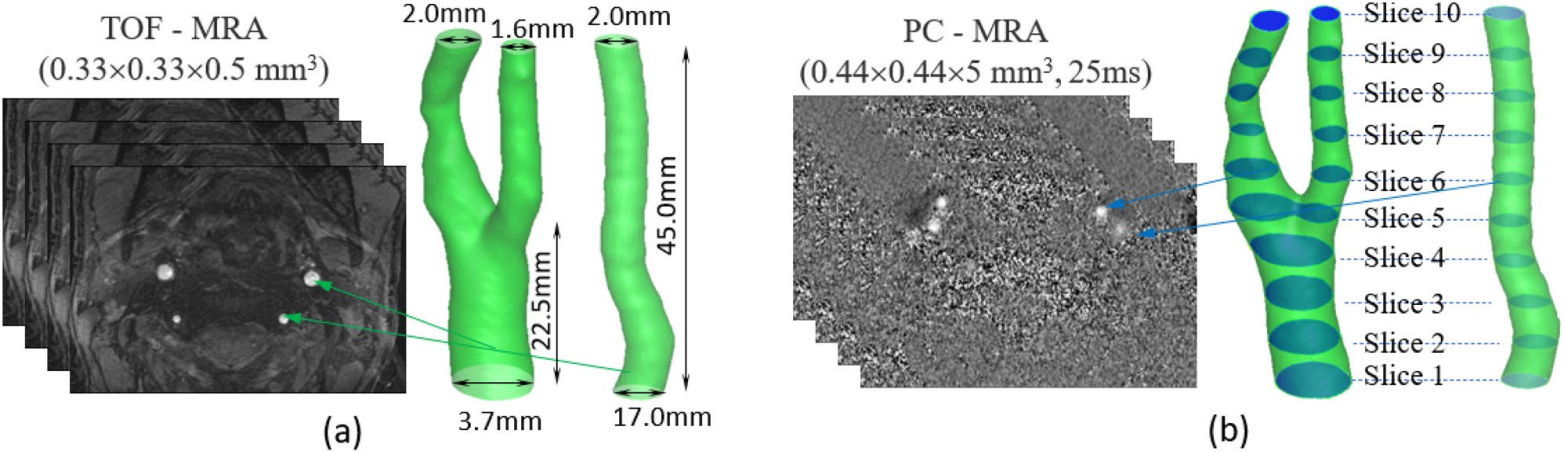
**(a)** High resolution 3-D TOF-MRA images and the segmented left carotid artery (with a bifurcation) and vertebral artery (tube-like). **(b)** 4-D Low resolution 4-D PC-MRA images with 10 locations along the arteries and 20 time points in one cardiac cycle.

**Figure 10:**
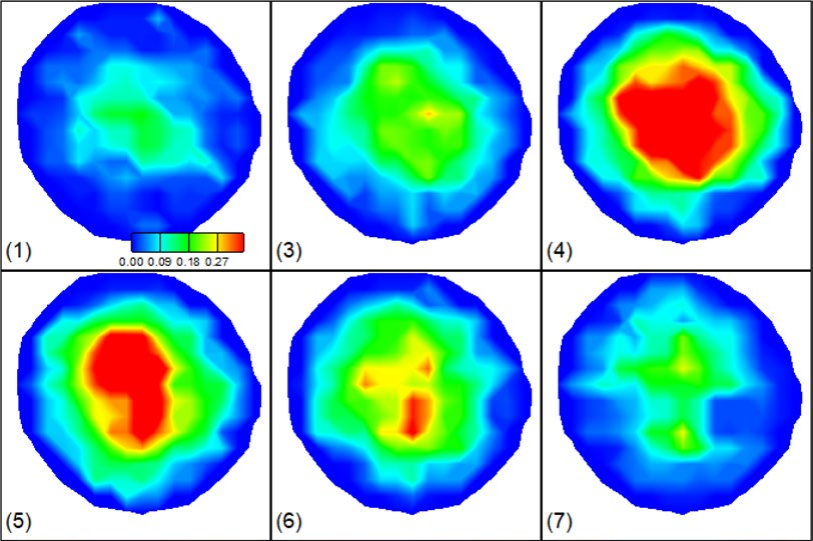
MRA-measured velocity magnitude contours on Slice 1 of the vertebral artery at 7 representative time points in a cardiac cycle. (4) corresponds the peak systole of the cycle.

The image data were acquired from the scanning of 3-D time-of-flight (TOF) and peripheral gated (PG) phase-contrast (PC) MRA on a 3T clinical MRI scanner (Siemens Magnetom Prisma) using a 64-channel head and neck coil. As shown in Fig. 9a, the TOF-MRA images with a high resolution of ${0.33}^{2}\times 0.5{\mathrm{mm}}^{3}$ are to anatomically extract the artery system. The TOF-MRA images are on DICOM data. In general, the image segmentation can be done by different software such as Materialise Mimics and 3D Slicer and exported in an STL format to follow the illustrated flowchart in Fig. 1. Since we have developed a method to do image segmentation from DICOM format and calculate the volumetric parameter $\mathcal{P}\left(\vec{x}\right)$ and the normal direction vector ${\vec{N}}^{w}\left({\vec{x}}_{b}\right)$ of each boundary cell^29,49^, the STL format is not necessary. We segmented the left carotid (with a bifurcation) and vertebral (tube-like) arteries, shown in Fig. 9a from the 3-D TOF-MRA images. The PC-MRA images in Fig. 9b, provide the 4-D velocity profiles on the cross-sections from the bottom up with a spatial resolution, ${0.44}^{2}\times 5{\mathrm{mm}}^{3}$ resulting in 10 slices in the vertical direction, and a time resolution of $25\mathrm{ms}$ resulting in 20 time points in one cardiac cycle. We use the in-house Matlab code to extract the 4-D velocity vector fields from the PC-MRA images for both vertebral and carotid arteries based on the method we developed^66^. Figure 10 shows the velocity magnitude contours on Slice 1 of the vertebral artery at 7 representative time points in one cardiac cycle. The 4th time point corresponds to the peak systole of the heart pumping. We simulated the blood flow in both carotid and vertebral arteries. The dimensions of the two arteries are found in Fig. 9a. The TOF-MRA resolution results in grid sizes of ${106}^{2}\times 416$ for the carotid artery and ${60}^{2}\times 416$ for the vertebral artery. The time resolution is 1 ms via linear interpolation of the PC-MRA data in Fig. 9b. The blood flow is driven by the 3-D velocity profiles at the inlet (Slice 1) and outlet(s) (Slice 10). We impose the non-equilibrium extrapolation scheme^61^ to introduce the velocity boundary conditions at the inlet and outlet(s). The density and kinematic viscosity of blood are $1.06\times {10}^{3} \mathrm{kg}/{\mathrm{m}}^{3}$ and $3.3\times {10}^{-6} \mathrm{m}/{\mathrm{s}}^{2}$, respectively. The relaxation time $\tau =0.505.$

**Figure 11:**
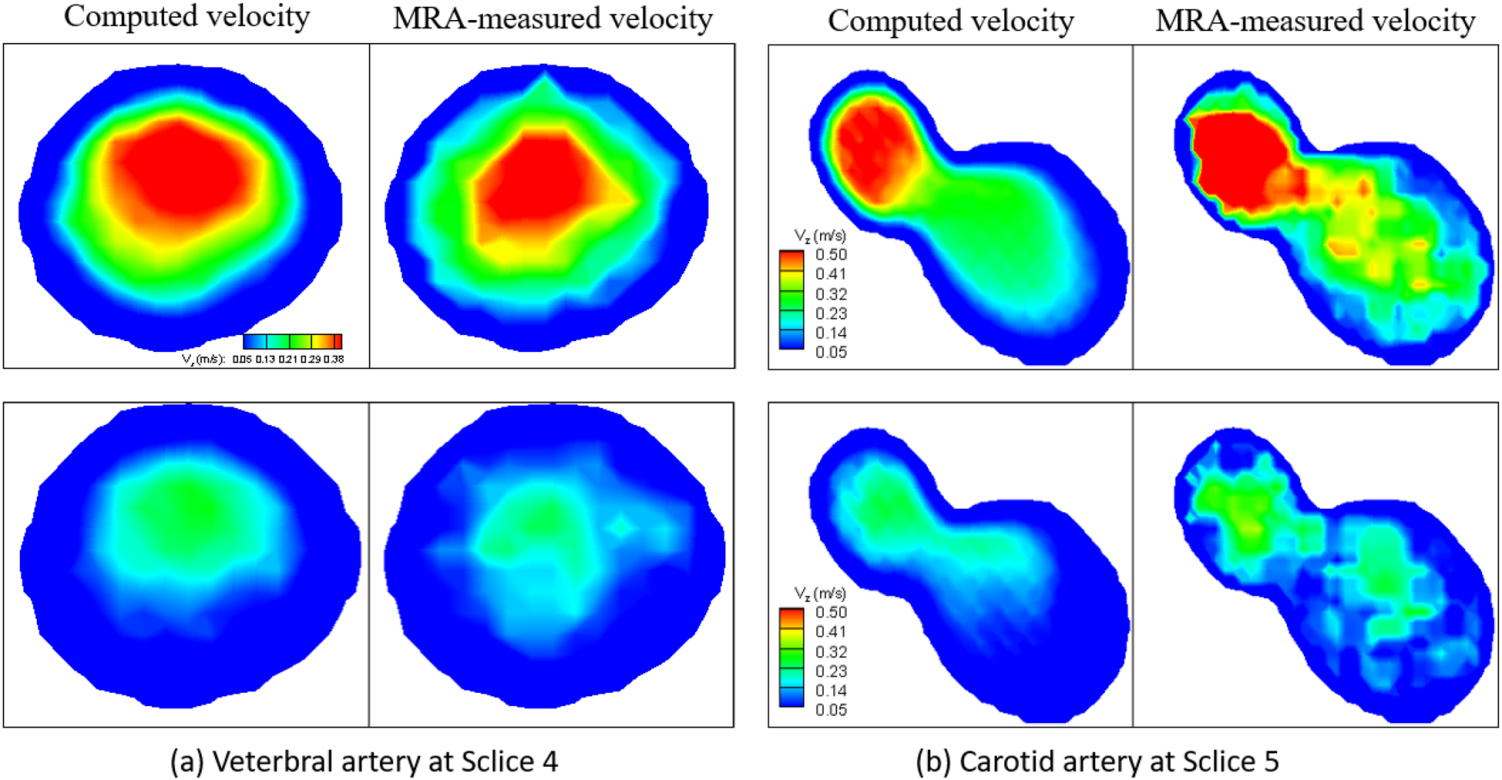
Comparisons of computed (left) vs. MRA-measured (right) velocity contours at **(a)** Slice 4 of vertebral artery at and **(b)** Slice 5 of carotid artery. On each panel, the top and bottom rows correspond to the peak systole and the end of diastole in a cardiac cycle.

**Figure 12:**
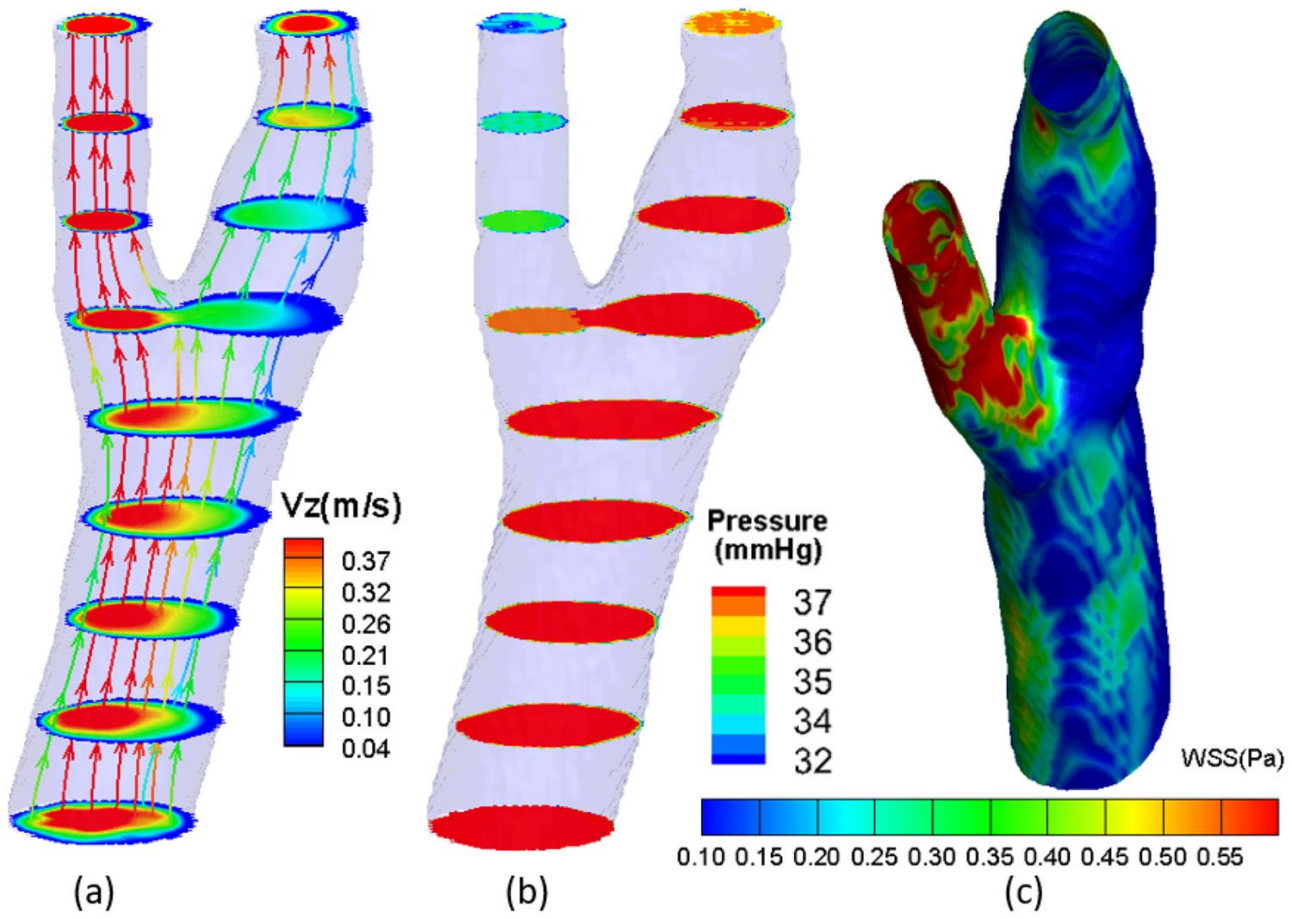
3-D **(a)** downstream velocity contours with streamlines, **(b)** pressure contours, and **(c)** of the carotid artery at peak systole.

We first show the comparisons of computed vs. PC-MRA measured velocity magnitude contours in Fig. 11 (a) vertebral (Slice 4) and (b) carotid (Slice 5) arteries, to demonstrate the accuracy of the computation. On each panel, the top and bottom rows correspond to the peak systole and the end of diastole in a heart beating. Overall, the computed velocity agrees with the MRA measured ones reasonably well. The MRA measured velocity is by nature noisy, appearing at both systole (large velocity) and end of diastole (small velocity.) Fig. 12 shows the 3-D (a) velocity contours with streamlines, (b) pressure contours, and (c) WSS distribution of the carotid artery at the peak systole. While the large velocity and WSS deviate to the internal carotid artery (left), the large pressure deviates to the external artery (right). This is due to the asymmetry between the internal and external carotid arteries. Such a reveal of the 4-D in vivo hemodynamics in live human arteries is meaningful to the patient-specific diagnostics and therapeutics for the advance of precision medicine. Further postprocessing of the computed 4-D velocity, pressure, and WSS will produce more medical relevant results, which will be included in a separate paper in near future.

## Summary and discussion

We have presented a unique computational method to quantify 4-D wall stresses of image-based pulsatile flows using the VLBM. With the introduction of a volumetric parameter to distinguish solid, liquid, and boundary cells, the VLBM is well-suited for dealing with complicated flow domains. The bounce-back boundary condition has been formulated in the streaming operation, thus avoiding complications of arbitrary boundaries. In this work, we have generalized the equation for the streaming operation from its original formation. The original streaming equation, Eq. (5), only accounts for streaming from a boundary cell to a fluid cell, which becomes problematic when the streaming is from a boundary cell to another boundary cell. We generalized Eq. (5), by introducing a parameter G, see Eq. (7), to distinguish two streaming scenarios and demonstrated the accuracy improvement. Starting from the provided image information, generally, in STL data format, we first construct the signed distance field based on the concept of level-set methods and directly calculate the volumetric parameter, $\mathcal{P}\left(\vec{x}\right)$, and the local wall normality, ${\vec{N}}^{w}\left(\vec{x}\right)$. The $\mathcal{P}\left(\vec{x}\right)$ and ${\vec{N}}^{w}\left(\vec{x}\right)$, together with inlet/outlet boundary conditions and initial conditions are directly fed to the VLBM, resulting in a seamless connection between the image processing and CFD. In the VLBM, the strain rate tensor can be calculated from the non-equilibrium particle population; therefore, the wall stresses can be obtained en-route during the VLBM implementation. These features, together with GPU parallel computing, dramatically reduce resource requirements for ICFD and ease the computational burden for pulsatile flows.

We applied the computational method to solve the benchmark pulsatile flows in an image-based pipe. We first simulated a Womersley (laminar) flow with Womersley number α and the base flow Reynolds number ${Re}_{0}$ of 6.89 and 9.4, respectively. The computational results were in good agreement with the analytical solutions, with average relative errors around 1.52% and 3.98% for mean downstream velocity and mean WSS, respectively. We found that the VLBM en-route calculation of WSS is more accurate than the 3-point centered FDMs. We further simulated turbulent flows by varying ${Re}_{0}$ from 535 to 4375 via direct numerical simulation. The computational results of velocity and WSS are again in good agreement with the experimental measurements. We have also presented one application study on the non-invasive quantification of 4-D hemodynamics, including velocity vector and pressure fields and the WSS distribution in MRA-based human carotid and vertebral arteries. The computed velocity agrees reasonably well with the MAR-measured velocity. Three more applications are currently active, including (1) quantification of hemodynamic WSS on image-based human choroidal vasculature endothelium, (2) Drag force on particles in sand flow, and (3) effects of waste streams on ion exchange kinetics in the porous structure. Application (1) is the first-ever computational quantification of WSS in the human choriocapillaris. The findings have been recently submitted for publication^67^. Applications (3) and (4) are in the code adaption stage and results are expected in near future.

Although static boundaries have been the focus in this work, the method can be easily generalized for moving boundaries as the next step. The original VLBM is derived for moving boundaries, and we have herein indicated how this might be applied. The formulation of volumetric LBEs consists of three parts: (1) collision taking into account the momentum exchange between the moving boundary and the flow; (2) streaming accompanying a volumetric bounce-back procedure in boundary cells; and (3) boundary-induced volumetric fluid migration moving the residual fluid particles into the flow domain when the boundary passes over a boundary cell toward a solid cell, and mass conservation is guaranteed in VLBM (no need to solve a pressure Poisson equation as in NS formulations). If time-varying image information is available, the presented computational method can be generalized to solve pulsatile flow problems in image-based moving boundaries.

## Data Availability

The datasets generated in the current study are available from the corresponding authors upon appropriate request for nonprofit use.
